# Social and behavioural factors in Non-suspicious unexpected death in infancy; experience from metropolitan police project indigo investigation

**DOI:** 10.1186/s12887-016-0541-x

**Published:** 2016-01-12

**Authors:** Andrew R. Bamber, Liina Kiho, Sam Upton, Michael Orchard, Neil J. Sebire

**Affiliations:** Department of Paediatric Pathology, Camelia Botnar Laboratories, Great Ormond Street Hospital for Children NHS Trust, Great Ormond Street, London, WC1N 3JH UK; Institute of Child Health, University College London, London, UK; Specialist Crime and Operations, SCO17, Metropolitan Police Service, London, UK; Department of Cellular Pathology, University Hospital of Wales, Cardiff, UK

**Keywords:** Infant death, SIDS, SUDI, Deprivation, Cosleeping

## Abstract

**Background:**

Risk factors for Sudden Unexpected Death in Infancy (SUDI) are well described, and such cases are now investigated according to standard protocols. In London, Project Indigo of the Metropolitan Police provides a unique, detailed framework for such data collection. We investigate such data to provide a contemporary account of SUDI in a large city and further link data to publically available datasets to investigate interactions with social factors.

**Methods:**

Retrospective analysis of data routinely collected by the Metropolitan Police Service in all cases of non-suspicious SUDI deaths in London during a six year period.

**Results:**

SUDI deaths are associated with markers of social deprivation in London. A significant proportion of such deaths are associated with potentially modifiable risk factors such as cigarette smoking and co-sleeping, such behaviour also being associated with social factors, including accommodation issues.

**Conclusions:**

Routinely collected data provide valuable insight into patterns and associations of mortality, with SUDI remaining a significant issue in London. Risk factors include social disadvantage, which may manifest in part by affecting behavioural patterns such as co-sleeping and public health interventions to reduce rates require significant social modification.

## Background

The United Kingdom has the highest all-cause mortality for children aged 0–14 in Western Europe, having a yearly excess of almost 2000 deaths in early life corresponding to >130,000 Potential Years of Life Lost (PYLL) for the country compared to countries with the lowest child mortalities [1]. Sudden Unexpected Death in Infancy (SUDI) is the single commonest group of post neonatal infant death in whom no pre-existing underlying medical condition is known [2]. In the United Kingdom (UK), SUDI cases are investigated on behalf of the Coroner, including autopsy performance by specialist paediatric pathologists, according to suggested guidelines [3]. In the Metropolitan area of London, non-suspicious infant and young child deaths, (under the age of two years for this protocol), include investigation by SCO17 (Specialist Crime and Operations; formerly SCD5), a specialist branch of the Metropolitan Police Service (MPS), according to standard protocol (Project Indigo), which collects data on a large number of variables relating to the circumstances of death, including social and behavioural factors of parents and carers [4]. The project Indigo dataset includes >140 fields in total and is completed on a proforma in all cases by trained officers. Cases of infant or childhood deaths in which there is evidence of associated crime (such as homicide or neglect) are investigated differently, including autopsy by specialist forensic pathologists, and are therefore not included in Project Indigo.

Previous epidemiological studies have identified numerous risk factors for SUDI, including young maternal age, social deprivation, smoking, cosleeping, and seasonal variation [2, 5]. Further understanding of the effects and interactions of such factors is important for development of effective public health and social policies. In England, the NHS Outcomes Framework (14/2014) provides key outcomes including Domain One (‘Preventing people from dying prematurely’) including ‘Potential Years of Life Lost (PYLL) from causes considered amenable to healthcare in children and young people’ (1aii) [2], and highlights reducing deaths in babies and children as an improvement area (1.6i) [2], shared with the public health outcomes framework (4.1) [6–8].

The aim of this study is to use a unique retrospective, descriptive dataset of non-identifiable records, derived from standard project Indigo investigation, of consecutive and unselected deaths in individuals under the age of two years in a well-defined urban geographical area in the United Kingdom, which includes the complete spectrum of social circumstance, linked with published markers of social deprivation as provided by the UK government, to examine the contemporary demographic features of SUDI, and specifically to examine the association between social and behavioural factors in such deaths.

## Methods

Routinely collected Metropolitan Police Service (MPS) Project Indigo data during a six-year period was reviewed (2005–2010 inclusive). All data were collected by specially trained police officers in SCD5 (now SCO17) according to a standard protocol. The dataset includes information regarding previous police contact (from the Police Database), and demographic and medical information provided by parents during a discussion with a specially trained police officer. Deaths are also categorised as medically explained, unexplained, or unascertained based on the pathologist’s opinion given in the postmortem report provided at the time of investigation. As these data were collected from a number of pathologists at different hospitals, and interpretation and use of terms in infant death is known to vary between practitioners, the term ‘unexplained’ in this context may not be directly equivalent to Sudden Infant Death Syndrome (SIDS) or SUDI. Records were linked to published markers of social deprivation [9] by the MPS data team and the final dataset was released for subsequent analysis in a fully anonymised format with no identifiers present. The use of data for this purpose was approved by the MPS (MO). Use of routinely collected autopsy data for research was also approved by the local research ethics committee (London (Bloomsbury) National Research Ethics Service Committee; formerly Great Ormond Street and Institute of Child Health Research Ethics Committee).

Descriptive analysis of the data was performed with particular regard to interactions of social deprivation markers and parent/carer behaviours. It is not possible to describe the full range of data collected in this manuscript, and this study focuses on risk factors for unexplained infant death highlighted in previous studies; specifically co-sleeping, lifestyle factors (such as alcohol and drug use), and social deprivation. Differences between groups were examined using comparison of proportion and chi-squared tests as appropriate, including chi-squared test for trend for ranked categorical variables (StatsDirect, UK).

## Results

During the period there were 477 deaths recorded in Project Indigo (2005 – 86, 2006 – 84, 2007 – 89, 2008 – 77, 2009 – 76, 2010 – 65). Overall, following post-mortem investigation, including full autopsy and ancillary investigations, 207/477 (43 %) were medically explained natural deaths, and 270 (57 %) remained unexplained, of which fourteen deaths (3 %) were classified as “Unascertained”. “Unascertained” is a term usually used to refer to deaths in which abnormalities were identified at autopsy which cannot be explained by the clinical history. Overtly suspicious deaths were not included in Project Indigo, and therefore the number of these cases occurring during the timeframe of reference is not available to us since these lie outside the dataset available.

**Fig. 1:**
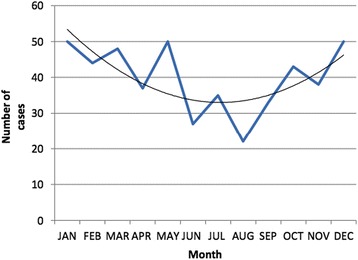
Number of deaths occurring each calendar month amongst 477 individuals aged less than two years dying in the Metropolitan police area between 2005 and 2010

**Fig. 2:**
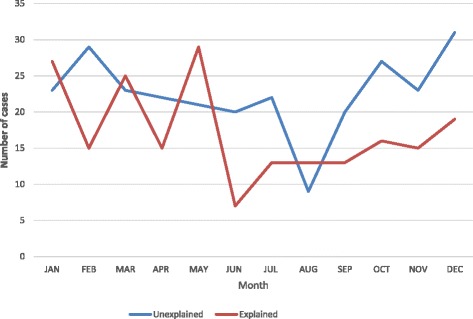
Number of deaths occurring each calendar month amongst 477 individuals aged less than two years dying in the Metropolitan police area between 2005 and 2010, divided into explained and unexplained deaths

Age data were available in all cases; 38 % were aged less than two months, 73 % below six months and 88 % below 1 year. Cases occurred throughout the year with peaks during the winter months (Figs. 1 and 2). The gestational age at birth was term in 297 cases (62 %), post-term (>42 weeks) in 14 (2.9 %) and preterm (<37 weeks’) in 117 cases (25 %); a significantly greater proportion than reported nationally [10] (8 %; *p* < 0.0002). Gestational age was not available in 49 cases. The proportion of natural vaginal births was significantly greater (53.4 % vs 41.8 %, *p* < 0.0002) and the proportion of assisted deliveries (instrumentation or induction) significantly lower, (12.6 % vs 33.8 %, *p* < 0.0002) than national data [10, 11]. Method of delivery was not recorded in 46 cases.

Data on behavioural risk factors are provided below. Alcohol was consumed regularly by 122/379 (32 %) of mothers in whom this information was available, whilst regular recreational drug use was reported in 34/383 (9 %). The use of alcohol and/or drugs on the day of death was reported in 64/355 (18 %) of mothers and 55/350 (16 %) of fathers. 40 % (163/406) reported maternal smoking of tobacco, and 41 % (159/386) reported paternal smoking of tobacco.

**Fig. 3:**
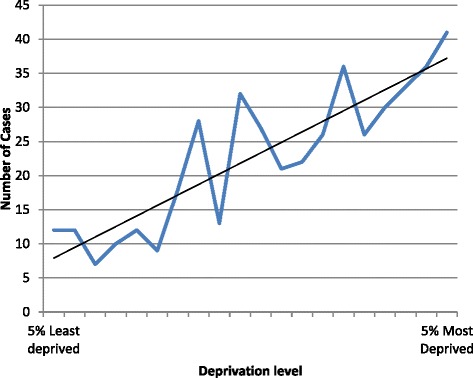
Deaths amongst 477 individuals aged less than two years dying in the Metropolitan police area between 2005 and 2010 arranged by Deprivation level (Indices of Multiple Deprivation Score Rank for Lower Layer Super Output Areas (LSOAs))

For each case, the individual postcode was linked to available indices of deprivation prior to anonymised data release (Indices of Multiple Deprivation Score Rank for Lower *Layer Super Output Areas* (*LSOAs*)). The number of deaths was significantly associated with increasing deprivation (Chi squared for trend 97.9; *p* < 0.0001; Fig. 3).

**Fig. 4:**
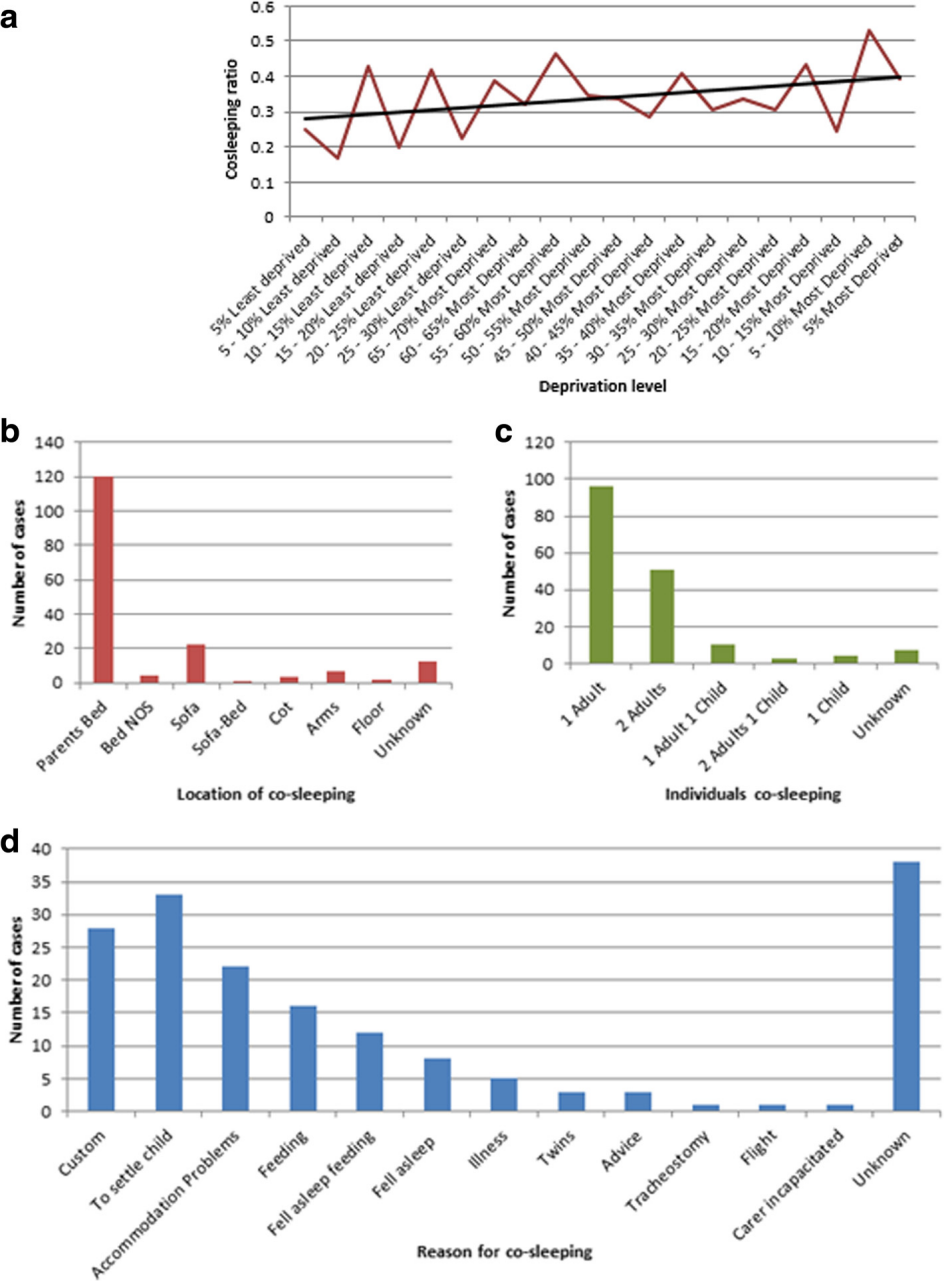
Characteristics of Co-sleeping on the occasion of death of 477 individuals aged less than two years dying in the Metropolitan police area between 2005 and 2010. **a** Co-sleeping ratio and deprivation: Deaths associated with co-sleeping in each Deprivation group (Indices of Multiple Deprivation Score Rank for Lower Layer Super Output Areas (LSOAs)) divided by the total number of deaths in that deprivation group. **b** Location of co-sleeping. **c** Individuals co-sleeping. **d** Reason given for co-sleeping

Thirty nine percent (171/441) of deaths were co-sleeping associated, accounting for significantly more of the unexplained than medically explained deaths during the period (50 %, 129/256 vs. 16 %, 34/207 respectively, *p* < 0.0002). There was a trend with greater levels of co-sleeping in more deprived areas. (Figure 4a) In the majority of cases, co-sleeping was with an adult in the parental bed but in 22 % (35/159) of cases co-sleeping was on a sofa, chair or other location known to be high risk (Fig. 4b and c). Where such information was provided, the reason for co-sleeping was related to accommodation problems in 17 % (22/133), the majority being related to either custom or in order to feed or settle the child (Fig. 4d).

## Discussion

The findings of this study have demonstrated that, first, numbers of SUDI deaths in London have remained relatively constant during the study period with around 40 % being medically explained following autopsy by specialist paediatric pathologists. Second, the findings confirm the previously reported seasonal variation in prevalence [5] and the peak age of 2–4 months [2]. Third, there is an association between rates of infant death and increasing levels of social deprivation. Fourth, regular alcohol and tobacco use are relatively common among parents/carers of affected children. Fifth, around 40 % of cases were co-sleeping-related deaths, representing approximately 50 % of medically unexplained cases. In the majority of these, co-sleeping was with an adult in the parental bed but in around 20 % it included co-sleeping on a sofa, chair or other known highly unsafe location.

The strengths of the current study are the unique dataset which was based on full ascertainment of consecutive unselected deaths from an urban geographic area in whom there was unified and standardised data collection for all cases by trained professionals (specialist Police officers) with no vested interest or bias in the future use of such data for research studies. All of the initial demographic analysis and data linkage to existing deprivation scores was performed independently by a statistical officer of the MPS (SU). The data are therefore highly representative of the population served and entirely unbiased. The data fields used represent factual responses to specific questions and do not involve any degree of interpretation of significance by the person or persons collecting such data.

There are some limitations to the data, meaning care must be used in its interpretation. First, there is a degree of variation in how practitioners may have use the terms SUDI, SIDS and unascertained, which is reflected in the post mortem report causes of death upon which the categorisation of death in this dataset is based. However, given that ONS (Office for National Statistics) data on causes of death is based on evidence given to the Coroner by the pathologist, there should be little practical difference between our dataset and ONS data. Second, as much of the data are self-reported by parents to police officers, it is possible that some fields may not be accurately reported, particularly those involving drugs of abuse and alcohol use. Finally, the dataset is composed of retrospective descriptive data and the results must be interpreted in that context.

Previous studies have examined the relationship between demographic factors and frequency of SUDI, which are confirmed by the current findings relating to contemporary infants deaths. For example, the age distribution, with a peak frequency of deaths at 2–4 months, and increased risk of death in infants born preterm are described [2]. The association with seasonality across all years of the study period, with more deaths during the winter months, has been reported but with variable findings [2, 5]. It has been suggested that the excess winter infant mortality may be related to respiratory infections in a significant proportion of such deaths [12]. A previous UK-based, population-based, case-control study including 325 infants dying without explanation and 72 infants dying of explained causes, in addition to matched controls, reported that there had been a recent reduction in the previously reported high winter peaks of death [13], although this is not supported by the present findings. The data in the present study appear to show a trend towards increased deaths in winter months amongst both explained and unexplained deaths. This is in keeping with the latest published data on unexplained infant deaths from the Office for National Statistics, which shows a continued trend of increased numbers of deaths in winter months [5].

The unique dataset used in the current study allows accurate linkage of deprivation score, as assessed by government derived multiple markers of deprivation, to other clinical and demographic features, with consequent demonstration of the strong association between areas with high deprivation scores and infant death rates. The association of adverse social factors and SIDS/mortality rates has long been recognised [2]. Based on parliamentary constituency data in Britain, and indices of deprivation, overall death rates across all ages were positively associated with deprivation score [14]. However, the additional findings from the present study allow assessment of the possible association between social deprivation and risk behaviour, such as smoking, alcohol or recreational drug use. It has previously been suggested that several epidemiological risk factors such as young maternal age and single parent family may not be significant once social deprivation factors have been accounted for, but parental smoking is associated with social deprivation markers and remains significant in multivariate analysis [15]. Similarly, in a large retrospective registry based study in Scotland, the infant death rate increased from the least deprived to the most deprived groups, and smoking was calculated to account for around 30 % of this difference. There are social variations in the effectiveness of health promotion campaigns, which may be attributable to differences in access to information and acceptability. For example, there was a rapid reduction in overall sudden infant death syndrome incidence during a period in the 1990s following the launch of the ‘back to sleep’ campaign in Scotland among women living in areas of relative affluence but with high deprivation, there was a much slower decline in death rates [16].

One of the unique strengths of the present study is to provide detailed data regarding co-sleeping associated deaths, in particular the circumstances of, and underlying reasons for, co-sleeping. The association between SUDI and cosleeping has been controversial, although it is now generally accepted that cosleeping of an infant with an adult in the first months of life is associated with increased risk of death if the adult is intoxicated or smokes [2, 17]. The current findings indicate that despite warnings regarding unsafe-sleeping environments for infants, around 20 % of parents/carers co-slept with their infants on a chair, sofa or other place; co-sleeping with infants on a sofa being associated with up to 40-fold increased risk of death [2]. In the majority of cases co-sleeping deaths were associated with behavioural factors, such as customary household practice (regardless of how often co-sleeping occurs), or as a means of feeding or settling the infant (i.e. a decision to co-sleep). Practical accommodation problems (i.e. leaving no alternative but to co-sleep) were a factor in an important, but relatively small number of cases. In this dataset, customary household practice is defined as generally accepted culture within the family unit, regardless of how often this occurs. These data suggest an association of co-sleeping associated deaths with increasing social deprivation. The findings are in keeping with a previous survey of urban caregivers, which reported that the majority co-slept, with greatest frequency in those who were single parents or with limited formal education [18]. Another study compared medical recommendations provided with actual parental practices and reported that families of higher socioeconomic group were both more aware of, and compliant with, medical recommendations for safe sleeping environments [19].

While this study has shown that co-sleeping is significantly more frequent in unexplained compared to medically explained deaths, the data also demonstrate that several associations, such as social deprivation and smoking, remain valid for all infant deaths, regardless of cause. This may indicate that some demographic risk factors represent a generalised increased risk for infant mortality, rather than any mechanisms specific to SIDS. A previous population-based, case-control study suggested risk factors for SIDS and explained SUDI are similar except for sleep position and breast feeding, with socioeconomic disadvantage and smoking being major risk factors for all [13]. In another study of all childhood deaths from registry data, there was an overall twofold higher mortality among the lowest compared with the highest socioeconomic categories, based on education, income, car access, and neighbourhood deprivation, which were strongest among infants but remained for all age groups and causes of death [20]. Based on data from all singleton live births in England and Wales for whom a deprivation score was assigned by postcode, deprivation had a strong effect on infant mortality, especially in the post neonatal period. It was estimated that one quarter of infant deaths could potentially be avoided if deprivation levels were reduced [21].

Potential weaknesses of the current study include that for some datafields no responses were provided despite completion of all relevant fields being mandatory. For example, specific data regarding usual alcohol consumption was not provided in 24 % of cases. In addition, parents and carers may have provided false answers to questions regarding factors such as alcohol and recreational drug use, particularly since the data were collected by police officers and there may be under-reporting of such factors. Additionally, since the data were fully anonymised prior to analysis for this study, no specific linkage to individual autopsy reports was possible (although the findings were used by the data team to assign final cause of death category), precluding assessment of specific autopsy findings with specific death circumstances. Such weaknesses are however inherent in all studies based on routine data collection, particularly relating to mandatory datasets collected on behalf of the police service. These weaknesses are offset by the complete ascertainment rate and uniformity and unbiased data collection methodology.

The current findings demonstrate that at least a proportion of the increased risk of infant death associated with social deprivation is potentially preventable, since they may be associated with risk behaviours by parents or carers. Important public health messages to reduce infant death rates include wider reduction of social disadvantage, but the present findings demonstrate possibility for more immediate and achievable reduction of risk behaviour. This has implications for health policies and interventions to target this high risk group, who may require different methods compared to non-deprived parents who are likely to read safe sleeping environment literature and take steps to reduce their risk. The more general association between ‘poor parenting’ and socioeconomic deprivation, including health inequalities has been well described [22], and parents living in deprived areas report increased parenting stress and concerns about behavioural and emotional problems of their children compared to those from less deprived areas [23]. Possible approaches therefore include both more simplified and universal advice regarding safe sleeping environments for infants, or interventions specifically targeted at this high risk parent group, requiring a coordinated approach by government and charities.

## Conclusions

In conclusion, this study, using a unique dataset, demonstrates continued association between infant death rate and social deprivation in a large urban population, and further provide data on the relationship between social disadvantage and increased parental/carer risk behaviours such as use of cigarettes and alcohol, and co-sleeping, which are potentially modifiable by public health interventions. Co-sleeping remains common in SUDI deaths and in most instances the reasons for co-sleeping are behavioural or cultural rather than practical. The findings highlight the difficulty and importance of effectively communicating public health messages around safe sleeping practices, particularly to high-risk groups and provide a baseline for future intervention or autopsy studies.

## Abbreviations

LSOAs: Layer Super Output Areas
MPS: Metropolitan Police Service
ONS: Office for National Statistics
PYLL: Potential Years of Life Lost
SCO17: Specialist Crime and Operations [formerly SCD5]
SIDS: Sudden Infant Death Syndrome
SUDI: Sudden Unexpected Death in Infancy
UK: United Kingdom
